# The Influence of the Social Support on Symptoms of Anxiety and Depression among Patients with Silicosis

**DOI:** 10.1155/2014/724804

**Published:** 2014-04-27

**Authors:** Bing Han, Bo Yan, Jian Zhang, Na Zhao, Jinkai Sun, Chao Li, Xibing Lei, Hongbo Liu, Jie Chen

**Affiliations:** ^1^Department of Health Statistics, School of Public Health, China Medical University, Shenyang, Liaoning 110001, China; ^2^Department of Pneumoconiosis, Shenyang No. 9 Hospital, Shenyang, Liaoning 110024, China; ^3^Department of Occupational and Environmental Health, School of Public Health, China Medical University, Shenyang, Liaoning 110001, China

## Abstract

The improvement of social support promotes the mental health and improves the health status. The study aimed to examine the influence of the social support on symptoms of anxiety and depression among patients with silicosis and provide the scientific basis to further alleviate anxiety and depression and to monitor their whole quality of life. We investigated 324 inpatients with silicosis between April 2011 and September 2011. The HADS (the Hospital Anxiety-Depression Scale) was the major methodology used to evaluate anxiety and depression, and the MSPSS (the Multidimensional Scale of Perceived Social Support) to evaluate the social support level. Among patients with silicosis, 99.1% had anxiety symptoms, and 86.1% had depression symptoms. Meanwhile, the social support significantly influenced symptoms of anxiety and depression. The study suggested that patients with silicosis presented more anxiety and depression symptoms, while the social support levels of the patients were relatively low. The influence of social support on symptoms of anxiety and depression among patients with silicosis implied that improving the level of social support and the effective symptomatic treatment might alleviate anxiety and depression symptoms and improve physical and mental status.

## 1. Introduction

Silicosis is a chronic, progressive, and fibrosing lung disease caused by inhaling respirable silica dust [1]. According to the report of U.S. National Institute for Occupational Safety and Health (NIOSH), the most important factor in the development of silicosis is “the product of the concentrations of dust containing respirable silica in workplace air and the percentage of respirable silica in the total dust” [2]. Currently, silicosis is a preventable but cureless occupational disease that no effective treatments for silicosis are available [3]. In 2007, the U.S. Occupational Safety and Health Administration (OSHA) estimated that more than two million employees were exposed to silica in general industry, construction, and maritime industry [4]. In 2000, more than 3.2 million workers were exposed in the European Union [5]. In China, 23812 new pneumoconiosis cases were reported in 2010, of which 9870 cases were silicosis, accounting for 41.45% of new pneumoconiosis cases [6]. Silicosis has become the greatest threat to workers' occupational safety and the most serious occupational disease worldwide.

Mental disorders of patients with silicosis need attention from physicians and scholars. The modern medicine not only focuses on the physical status of patients but also concerns about the mental status of patients. The good mental status of patients complies with the procedures of medical interventions, achieves benefits of more accurate diagnostics and more efficient therapeutic interventions, and helps to cope with the progression of diseases [7]. In particular, the mental status of patients crucially relates with silicosis. Due to illness or frequent hospitalization, patients with silicosis were prone to anxiety and depression [8]. In Turkey, sandblasters' silicosis increased anxiety levels and caused depressive symptoms [9]. In China, patients with silicosis had higher prevalence of depressive symptoms [10]. The presence of anxiety and depression associated with deteriorating physical health and impairing quality of life [11, 12].

Social support is widely perceived as a positive factor on mental health. It is defined as the process “through which the social relationships promote health and well-being” [13]. Social support relieves stress, and positive social support plays an important role to improve quality of life [14]. It was reported that the lack of quality social support, depression, and social isolation led to problems such as heart diseases or breast cancer [15–17]. Social support is provided by networks consisting of family, relatives, friends, neighbors, and coworkers, especially when the interaction is positive [18]. Social support serves as the major source in decreasing negative psychological reactions such as hopelessness and depression and helps to decrease harmful effects of negative events in life on physical health and emotional well-being and buffers when coping with stress [16, 17]. Hence, the improvement of social support helps to promote the mental status and further improve the whole quality of life.

The main aim of the study was to investigate whether social support would help to promote the mental status of patients with silicosis and further improve their whole quality of life. Therefore, in this study, we measured social support, symptoms of anxiety, and depression of patients with silicosis, explored the influence of the social support on symptoms of anxiety and depression among patients with silicosis, and found related factors which might influence anxiety and depression symptoms of patients with silicosis. The present study was to provide the scientific basis to further relieve anxiety and depression symptoms and to monitor their whole quality of life.

## 2. Materials and Methods

### 2.1. Design and Subjects

We collected data on inpatients with silicosis according to hospital records of the Shenyang No. 9 Hospital (18 South 11th West Road, Shenyang, China) between April 2011 and September 2011. All enrolled patients were able to think and answer questions clearly. Face-to-face interviews were conducted by two experienced and trained interviewers using structured questionnaires, and most patients were interviewed separately. Interviews of patients who had difficulty in understanding were completed in the ward.

The diagnosis of silicosis was based on Chinese diagnostic criteria of pneumoconiosis and corresponding standard films of pneumoconiosis and was made by five qualified experts, who were all members of the Shenyang Municipal Pneumoconiosis Diagnosis Committee, with the records of historical exposure to silica and chest X ray. The definition of I period silicosis is that the total profusion of small opacities is grade 1 and the distribution of range reaches at least two lung zones. The definition of II period silicosis is that the total profusion of small opacities is grade 2 and the distribution of range reaches more than four lung zones, or that the total profusion of small opacities is grade 3 and the distribution of range reaches four lung zones. The definition of III period silicosis is that the total profusion of small opacities is grade 3 and the distribution of range reaches more than four lung zones, or that the large opacities is at least 20 *μ*m long and 10 *μ*m wide.

The research protocols were reviewed and approved by the hospital's ethical committee group. Patients that volunteered for the study fully understood the study purpose and were guaranteed anonymously. No patients without verbal consent were enrolled to this study.

### 2.2. Measurements

Data collected in this study included demographic characteristics (age, gender, marriage, etc.), smoking status (no smoking and smoking), drinking status (no drinking and drinking), dust exposure history (name and type of company, job title, age at first exposure, duration of dust exposure, etc.), and clinical characteristics related to silicosis (date of diagnosis, period of disease, comorbidity, etc.).

### 2.3. The Multidimensional Scale of Perceived Social Support

The Multidimensional Scale of Perceived Social Support (MSPSS) is used to measure patients' perceptions of social support from three specific sources: family, friends, and significant others. The MSPSS which demonstrates good internal reliability and adequate temporal stability was used to evaluate the social support status, and strong factorial validity and moderate construct validity were also demonstrated in the study [19]. The Chinese version (MSPSS-C) has demonstrated high internal consistency with a Cronbach's alpha equal to 0.89; therefore, its validity and reliability are confirmed [20]. It uses a 7-point Likert Scale set from 1 (strongly disagree) to 7 (strongly agree). The total score ranges from 12 to 84 [21]. Higher scores indicate higher perceptions of social support and lower scores indicate lower perceptions of social support [21].

### 2.4. The Hospital Anxiety and Depression Scale

We applied the Chinese version of the Hospital Anxiety and Depression Scale (HADS), a widely used instrument that is developed to evaluate anxiety and depression occurring in chronic diseases [22], to evaluate anxiety and depression occurring in patients with silicosis. The HADS has previously demonstrated the sensitivity above 80% and the specificity above 90% in a cohort of elderly Chinese patients [23]. The HADS consists of seven items for anxiety (HADS-A) and seven for depression (HADS-D). Items are scored on a four-point scale from zero (not present) to three (considerable) and scores are added, given subscale scores on the HADS-A and the HADS-D from 0 to 21 [24]. In this study, a score above or equal to 8 on either subscale is conventionally used to define anxiety and depression [25].

### 2.5. Statistical Analysis

The duration of dust exposure for each patient was calculated by the accumulation of durations of all jobs with dust exposure. The comorbidity was quantified as the numbers of associated diseases. Personal income was divided into two groups: low (<1500 RMB per month) and high (≥1500 RMB per month). Quantitative variables that included age and duration of dust exposure were described as mean ± standard deviation. The qualitative variables, which included gender, marital status, personal income, education level, smoking status, drinking status, period of silicosis, pulmonary tuberculosis, and number of comorbidity, were described as the absolute number or proportions. The *t*-test was used to compare the social support, anxiety, and depression of patients with silicosis in different stages of age, gender, period of silicosis, and pulmonary tuberculosis. The one-way ANOVA was used to compare the social support, anxiety, and depression of patients with silicosis in different stages of duration of dust exposure. Partial correlation coefficients and hierarchical multiple linear regression models were calculated to examine the association between social support and anxiety and depression of patients with silicosis.

The study applied the hierarchical multiple linear regression models to explore the influence of the social support on symptoms of anxiety and depression among patients with silicosis. Therefore, anxiety or depression served as outcome variables in the models. In the progress, the first block (Model I) established that social support was included as predictor variable. The second block (Model II) in models added period of silicosis, duration of dust exposure, and pulmonary tuberculosis as covariates to further explore the influence of the social support on symptoms of anxiety and depression among patients with silicosis. The third block (Model III) demonstrated other known covariates including age, gender, marital status, educational level, smoking status, and drinking status. The fits of three blocks were all assessed using the adjusted *R* ^2^-value. *P* < 0.05 (two-sided) was considered statistically significant. All statistical analysis was performed using SPSS 12.0 (SPSS, Chicago, IL, USA).

## 3. Results

### 3.1. Basic Characteristics

The 324 inpatients with silicosis enrolled in the present study were mainly exposed to silica dust in their workplaces. Their mean age was 74.1 (SD 10.1) (range from 36 to 91), and 251 (77.5%) of them were men, while 73 (22.5%) were women. The average duration of dust exposure was 27.9 (SD 8.5) (range from 2 to 44) years. There were 282 (87.0%) I period silicosis, 29 (9.0%) II period silicosis, and 13 (4.0%) III period silicosis [26].

### 3.2. Comparison of Social Support, Anxiety, and Depression of Patients with Silicosis in Related Variables

Table 1 showed that the mean score of social support was 55.3 (SD 10.3), HADS-A was 12.9 (SD 1.9), and HADS-D was 10.5 (SD 1.9). Based on the predefined cut-off points, 321 (99.1%) patients had anxiety and 279 (86.1%) patients had depression.

Patients over 60 years old with silicosis had significantly lower scores of social support (*t* = 3.627, *P* < 0.001). Female patients had significantly lower scores of social support (*t* = 4.317, *P* < 0.001) and higher scores of depression (*t* = 4.623, *P* < 0.001) than male. The patients with 16–30 years of dust exposure had significantly the lowest scores of social support (*F* = 3.796, *P* = 0.023) and highest scores of depression (*F* = 9.063, *P* < 0.001). At the same time, patients with more than 30 years of dust exposure had significantly highest scores of social support (*F* = 3.796, *P* = 0.023) and lowest scores of depression (*F* = 9.063, *P* < 0.001).

### 3.3. The Influence of the Social Support on Symptoms of Anxiety and Depression among Patients with Silicosis

After adjustment for age, gender, personal income, education level, duration of dust exposure, and number of comorbidity, the partial correlation coefficient of social support and anxiety was −0.113 (*P* = 0.044). It suggested that anxiety symptoms of patients with silicosis might be alleviated with increasing social support. After adjustment for gender, education level, duration of dust exposure, and number of comorbidity, the partial correlation coefficient of social support and depression was −0.373 (*P* < 0.01). It suggested that depression symptoms of patients with silicosis might be alleviated with the improvement of social support.

Table 2 showed that social support was significantly influenced on anxiety symptoms of patients with silicosis in Model I. This association was also significant in Model II, in which period of silicosis, duration of dust exposure, and pulmonary tuberculosis were included as adjusting covariates. With period of silicosis, duration of dust exposure, and pulmonary tuberculosis added in the model increased the explained variance by 3.6%. Personal income was also significantly associated with anxiety (Model III), and the model improved by 10.8% with age, gender, marital status, educational level, smoking status, and drinking status added in the model. It also suggested that anxiety symptoms of patients with silicosis might be alleviated with increasing social support.

The same pattern was found when the depression symptoms of patients with silicosis were used as an outcome variable in the Table 3. Social support and depression of patients with silicosis had a similar association as anxiety, and the model was explained to 37.3% (Model I). While the model was adjusted with period of silicosis, duration of dust exposure and pulmonary tuberculosis (Model II), and social support were significantly influenced by depression symptoms of patients with silicosis and the explained variance increased by 2%. Age was significantly associated with depression symptoms as well (Model III). However, the change in explained variance was minor (5%). It also suggested that depression symptoms of patients with silicosis might be alleviated with the improvement of social support.

## 4. Discussion

In this study, we measured the levels of social support, anxiety, and depression of patients with silicosis in China. We found that 99.1% patients with silicosis had anxiety symptoms, and 86.1% patients with silicosis had depression symptoms, while the social support level of them was relatively low as a previous study showed that scores in mid-50s indicated low level of social support [27]. Compared with the rates of anxiety and depression symptoms of other chronic diseases such as diabetes [28], the results indicate that patients with silicosis have more severe anxiety and depression symptoms. Other related studies also confirmed that the elderly patients with silicosis had high rates of anxiety and depression symptoms [10, 29].

We also found that there were negative correlations between levels of social support and symptoms of anxiety and depression. It suggests that social support would alleviate symptoms of anxiety and depression among patients with silicosis. In China, social support of patients with silicosis mainly comes from employee compensation, general insurance, families, and friends [30]. Employee compensation was the basic economic resource of the patients with silicosis, while insurance covers their basic medical costs of the patients with silicosis. Families and friends of patients with silicosis provide social support and relieve their psychological stress through daily life and communication. The personal networks that provide social support maintain emotional well-being and buffer the effect of adverse life events, and it has a direct and independent effect on mental health irrespective of presence or absence of stressful life events. Uchino believed that social support affected health by regulating psychological mood and facilitating physical activities [31]. Therefore, higher levels of social support can alleviate anxiety and depression symptoms of patients with silicosis. However, due to the low level of social support of patients with silicosis, the rate of anxiety and depression symptoms of them is likely higher, which suggests higher psychological stress of patients with silicosis. Therefore, improving the level of social support of patients with silicosis may alleviate their anxiety and depression symptoms. Furthermore, effective psychotherapeutic interventions should be considered to solve psychological disorders in patients with silicosis, which include the cognitive behavioral therapy, the acceptance and commitment therapy, and individual interpersonal psychotherapy [32, 33].

An additional finding is that education level is also significantly associated with anxiety symptoms of patients with silicosis. Hicks and Heastie found that high education level heightened anxiety [34]. Patients with higher education level are either supervisors or more skilled workers. Their employee compensation is higher than patients who have low education level. Patients with low education level usually are satisfied with basic medical and live services with moderate income, but patients with high education pay more attention to their physical and mental status by their relatively higher income, and they desire better medical and live services, which may make them more psychologically stressful. It may heighten the level of anxiety of patients with silicosis, thereby necessary psychotherapeutic interventions should be taken into account.

Our findings also reveal that age is significantly associated with depression symptoms of patients with silicosis, while a previous study showed elderly patients with pneumoconiosis from Hong Kong who also reported more depression symptoms [29]. Although the physical status of patients over 60 years old is relatively worse than those less than 60 years old, they are easier to be satisfied as their demands to the quality of life are relatively lower than those less than 60 years old. In contrast, the patients less than 60 years old are more anxious for their physical and mental status. These may be related to their psychological disorders. Such disorders might lead to their depression symptoms. The results register that patients with silicosis need positive symptomatic treatment and more psychotherapeutic interventions.

The main limitation of this study was that it was a cross-sectional study. The occupational history data were not investigated detailedly since the employer of enrolled patients had been bankrupt or relocated to the outer suburbs of Shenyang. So the results of patients with silicosis with multiple occupational history were not analyzed.

## 5. Conclusions

In the present study, patients with silicosis present more anxiety and depression symptoms, while the social support level of patients with silicosis is relatively low. The study shows the social support influences on symptoms of anxiety and depression among patients with silicosis. Improving the level of social support of patients with silicosis will alleviate their anxiety and depression symptoms and enhance their health status.

## Figures and Tables

**Table 1:** Comparison of the social support, anxiety, and depression of patients with silicosis in related variables.

Variables	*n*	Social support	Anxiety	Depression
Age				
(0) ≤60	39	60.7 ± 8.8	13.0 ± 2.0	10.6 ± 2.0
(1) >60	285	54.5 ± 10.3*	12.8 ± 1.9	10.5 ± 1.9
Gender				
(0) Female	73	50.8 ± 9.9	12.9 ± 2.0	11.4 ± 1.9
(1) Male	251	56.5 ± 10.0*	12.8 ± 1.9	10.3 ± 1.8*
Period of silicosis				
I	282	55.2 ± 10.4	12.9 ± 1.9	10.5 ± 1.8
II-III	42	55.5 ± 9.3	12.6 ± 1.8	10.7 ± 2.1
Duration of dust exposure				
(0) ≤15	32	54.4 ± 10.9	12.5 ± 1.6	10.3 ± 1.4
(1) 15–30	172	54.0 ± 10.1	12.9 ± 1.9	10.9 ± 1.9
(2) >30	120	57.3 ± 10.2^#^	12.9 ± 2.0	10.0 ± 1.8^#^
Pulmonary tuberculosis				
(0) Yes	130	55.9 ± 9.0	12.7 ± 1.9	10.4 ± 1.8
(1) No	194	54.8 ± 11.0	13.0 ± 2.0	10.6 ± 1.9
Total	324	55.3 ± 10.3	12.9 ± 1.9	10.5 ± 1.9

*Compared with (0), *P* < 0.05; ^#^compared with (1), *P* < 0.05.

**Table 2:** Hierarchical regression models for the influence of the social support on anxiety symptoms among patients with silicosis.

Anxiety	Model I	Model II	Model III
	*B* (SE)	*B* (SE)	*B* (SE)
Social support	−0.0012 (0.0005)*	−0.0014 (0.0005)**	−0.0016 (0.0005)**
Period of silicosis	—	−0.282 (0.328)	−0.202 (0.330)
Duration of dust exposure	—	0.169 (0.172)	0.185 (0.180)
Pulmonary tuberculosis	—	−0.190 (0.225)	−0.014 (0.233)
Age	—	—	0.001 (0.015)
Gender	—	—	−0.183 (0.299)
Marital status	—	—	0.268 (0.263)
Personal income	—	—	0.286 (0.255)
education level	—	—	0.610 (0.245)*
Smoking status	—	—	0.241 (0.235)
Drinking status	—	—	−0.252 (0.290)
Adjusted *R* ^2^	0.104	0.140	0.248
Adjusted *R* ^2^ change	—	0.036	0.108

*P < 0.05; **P < 0.01.

**Table 3:** Hierarchical regression models for the influence of the social support on depression symptoms among patients with silicosis.

Depression	Model I	Model II	Model III
	*B* (SE)	*B* (SE)	*B* (SE)
Social support	−0.068 (0.009)***	−0.065 (0.009)***	−0.061 (0.010)***
Period of silicosis	—	0.335 (0.293)	0.441 (0.295)
Duration of dust exposure	—	−0.295 (0.154)	−0.152 (0.160)
Pulmonary tuberculosis	—	−0.256 (0.201)	−0.093 (0.208)
Age	—	—	−0.740 (0.267)**
Gender	—	—	−0.020 (0.013)
Marital status	—	—	−0.254 (0.235)
Personal income	—	—	−0.012 (0.219)
education level	—	—	−0.012 (0.228)
Smoking status	—	—	0.320 (0.209)
Drinking status	—	—	−0.254 (0.259)
Adjusted *R* ^2^	0.373	0.393	0.443
Adjusted *R* ^2^ change	—	0.020	0.050

**P < 0.01; ***P < 0.001.
